# The Effect of Niobium Addition and Pre-Annealing on the Tensile Properties of 52CrMoV4 Spring Steel

**DOI:** 10.3390/ma17030583

**Published:** 2024-01-25

**Authors:** Arzu Ozuyagli, Zafer Barlas, Ugur Ozsarac, Suleyman Can Kurnaz

**Affiliations:** 1 Olgun Celik, Hasan Turek Bulvari, 45030 Manisa, Türkiye; 2 Metallurgical and Materials Engineering Department, Faculty of Technology, Sakarya University of Applied Science, 54050 Sakarya, Türkiye; barlas@subu.edu.tr (Z.B.); ozsarac@subu.edu.tr (U.O.); 3 Metallurgical and Materials Engineering Department, Faculty of Engineering, Sakarya University, 54050 Sakarya, Türkiye; ckurnaz@sakarya.edu.tr

**Keywords:** leaf spring material, niobium content, preheating, 52CrMoV4 spring steel, hot rolling process, microstructure, mechanical property

## Abstract

In this study, the effect of niobium addition and a specific preheating process on the microstructure and tensile properties of 52CrMoV4 steel used in leaf springs was investigated. Flat and leaf spring materials were used to accomplish this aim. The flat materials under investigation were kept in a furnace for 90 min at 900 °C. A homogeneous microstructure was aimed for with the use of this pre-annealing heat treatment in addition to the standard process before rolling used to create NbC. Leaf spring production was carried out with flat materials that possessed various Nb contents, with or without pre-heating. Grain size measurement and tensile tests were performed on the flat and leaf springs. Additionally, scanning electron microscopy images were captured from the fractured surfaces after the tensile tests were carried out. The current study highlights the importance of Nb addition as an alloying element and the effect of the selected pre-annealing process in optimizing the grain structure and enhancing the tensile properties of leaf springs. The leaf spring with a Nb ratio of 0.0376 that was pre-annealed exhibited a finer grain structure (G = 11.3), greater tensile properties (YS = 1550 N/mm^2^ and UTS = 1688.6 N/mm^2^), and deeper tear valleys and larger dimples, indicating higher energy consumption during fracturing, according to the SEM images produced, in contrast with the other materials studied.

## 1. Introduction

In the automotive industry, where competition has increased considerably in recent years, suspension systems are of great importance in terms of both driving comfort and driving safety. One of the most important components of the suspension system is the springs. Leaf springs are one of the most important and critical suspension components in terms of providing the connection between the axle and chassis and making the ride more bearable against impacts from the road. Leaf springs are traditionally manufactured from different materials such as 55Cr3, 51CrV4, 52CrMoV4, 51B60H, and 5160H, which are plate steel materials [1]. There are various types of leaf springs based on the vehicle, its tonnage, and the axle of the vehicle on which they will be utilized. There are numerous different types, including multi-layer conventional leaf springs, air bellow springs, and parabolic leaf springs. The parabolic leaf springs are a key suspension component that add significantly to the lightening of the suspension system by delivering spring characteristics and strength properties while using the fewest layers. A typical steel leaf spring has extra weight, which influences fuel efficiency. For this reason, tensile properties become more important in single-layer parabolic leaf springs [2].

Heat treatment can be applied to the leaf spring material in order to improve its mechanical properties. Since the mechanical properties specified in the relevant raw material standard are insufficient in a part that can be exposed to dynamic forces, it is very important to apply heat treatment, especially in parts that are exposed to constantly changing load conditions, such as leaf springs. In the production of leaf springs, the spring steel is annealed at 800–1000 °C, depending on the thickness of the material, and cooled in oil. The interior structure turns martensitic at this point. Since the martensitic structure is brittle, a tempering process is carried out in order to obtain a material with higher fracture toughness and better mechanical properties. The cooled semi-finished products are tempered between 450 and 550 °C according to the hardness of the annealing and left to cool at the outdoor temperature with water supplementation. As a result, the material gains high elasticity properties and mechanical properties that reach the desired level [1].

52CrMoV4 is a high-strength, low-alloy steel that is commonly used in heavy-duty applications, such as crankshafts, gears, springs, and connecting rods. Leaf springs play a crucial role in providing reliable suspension systems for various vehicle applications. These suspension components must exhibit superior mechanical properties to withstand demanding operating conditions, including heavy loads and dynamic forces. As a result, researchers and engineers continuously seek innovative approaches to enhance the performance of these critical components [3]. Atakay et al. examined the changes in the internal structure and, therefore, mechanical properties of a hot-rolled ribbed wire rod steel with the addition of Nb (through microalloying). It was observed that Nb precipitates in the grain, both refining the grain structure (providing grain refining) and increasing the hardness through its strong carbide-forming manner. By adding low amounts of Nb to the steel, the yield and tensile strength values were significantly increased. Additionally, an increase in the ductility and toughness of the steel was also achieved [4].

Qin et al. investigated the effect of niobium alloying on the fatigue properties of 50CrV leaf spring material. As a result, it was observed that the Nb additive increased the tensile strength. The strengthening effect of the NbC and NbN(C) particles precipitated during the process and after the phase change improved the strength level [5]. Zhang et al. investigated the effect of adding 0.022 wt.% Nb to 60Si2MnA spring steel on the austenite grain size and decarburization. By the end of the study, it was determined that the austenite grain size had become smaller, and the decarburization depth decreased with the addition of niobium content [6]. Klinkenberg et al. studied the effects of adding niobium as an alloying element to steels in automotive applications. The grain refinement effect of niobium is mainly due to the delay or prevention of recrystallization in the final hot-forming steps. It is mentioned in the study that in order to make the most of its metallurgical potential, annealing should be performed before hot working to dissolve the Nb(C,N) [7].

The effect of the ratio of niobium as an alloying element on the tensile properties of 52CrMoV4 steel material has not been investigated in previous studies. However, there are studies on the effects of Nb addition to other alloy steels. Zhu et al. observed that Nb, as an alloying element, was effective at increasing the strength and toughness by delaying recrystallization and reducing the grain size [8]. In a study carried out by Zakir, niobium was found to have the highest grain refinement effect among all alloying elements studied. In the study, it was proven that the limit for Nb addition is 0.04 wt.%. It was observed that the toughness of the spring steel was at the maximum level with 0.04 wt.% Nb as the alloy element [9]. Ghanaei et al. studied the effect of adding different amounts of vanadium and niobium microelements to different types of steel after bake hardening. It was found that the highest tensile strength value and finest grain size were achieved in the sample with 0.031 wt.% Nb [10]. According to Andrade et al., Nb has the greatest solute retardation effect on static recovery and recrystallization. In their study, the authors found that recrystallization was delayed by the precipitation of NbC during hot processing [11]. Akthar et al. investigated the non-recrystallization temperature (TNR) of Nb-added micro-alloyed steel. This study showed that the value of the recrystallization temperature determined for 0.1 wt.% niobium micro-alloyed steel is ~1000 °C [12].

It is commonly known that when a niobium alloying element is added to steel, it has a grain size reduction effect. In this study, the effect of the Nb alloying element and pre-annealing process on the tensile properties of parabolic leaf spring steels was investigated, and 52CrMoV4 steel used in leaf spring production was selected as the raw material. Niobium as an alloying element was added in two different ratios to this type of steel.

One such approach involves investigating the effects of alloy composition and heat treatment parameters on the mechanical properties of spring steels. In this context, the present article delves into the examination of two vital factors: the niobium content and the pre-heating process in relation to the mechanical properties of the 52CrMoV4 spring steel alloy after hot deformation.

The standard parabolic leaf spring production process is as follows: it consists of cutting, drilling, hot rolling, annealing, bending, tempering, shot-peening, surface treatments, and assembly steps. The aim of this process is to obtain a more homogeneous structure, with the pre-annealing heat treatment in addition to the standard process before rolling to create NbC carbide, which makes the grain size finer; to investigate whether a material with a smaller grain size is formed after rolling via its stable carbide structure; and to investigate the effect of this process on the tensile properties of the spring samples. In order to investigate the mechanical properties of niobium alloy elements and pre-annealing processes in parabolic leaf springs, the microstructure and strength properties of the above-mentioned materials were examined. The aim of this investigation was to provide additional information regarding the materials that contribute to the general efficiency and safety of vehicles. This study highlights the importance of Nb addition as an alloying element, in addition to the effect of the pre-annealing process, in optimizing the grain structure and tensile properties of leaf spring materials.

## 2. Materials and Methods

In this study, 52CrMoV4 spring steel was used. The nominal composition of this grade according to DIN EN 10089 [13] and the chemical analysis of the supplied flat materials are shown in Table 1. The flat materials obtained for the experimental studies were produced by the continuous casting process at the ÇEMTAŞ Steel Factory, Bursa, Türkiye.

**Figure 1:**
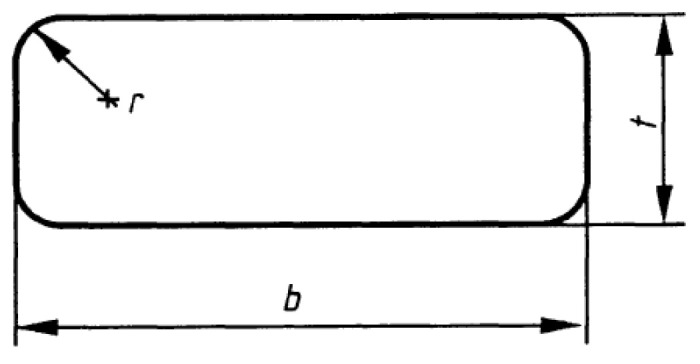
Flat material cross-section geometrical details (EN 10092).

**Figure 2:**
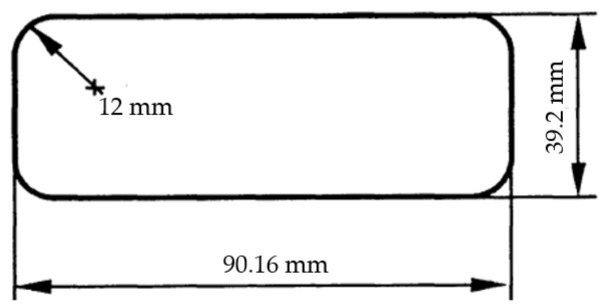
Section measurement of the flat material (FM).

This study obtained flat material from the raw material supplier to produce single-layer leaf springs. The dimensions of the obtained flat material were produced in accordance with the Form C (EN 10092) standard [14]. The flat material cross-section details are provided in Figure 1. The flat material was coded as FM. The thickness (t) was determined to be 39 mm, the radius (r) was determined to be 12 mm, the width (b) was determined to be 90 mm, and the length was determined to be 1670 mm. The flat material cross-section measurements are shown in Figure 2.

The study aimed to conduct a comparison by producing pre-annealed and non-pre-annealed versions of these raw materials. The FMs were kept in the furnace for 90 min at 900 °C. When the samples were taken out of the oven and their temperatures were recorded, it was found that the whole surface had reached 900 °C. The hot rolling process was carried out after the samples were kept at room temperature for 24 h.

The production of parabolic leaf springs was carried out using four different materials, as indicated in Table 2. The first material is 52CrMoV4 spring steel without pre-annealing and with a Nb ratio of 0.0042, which is coded as AN. The second material is 52CrMoV4 spring steel, which was pre-annealed and has a Nb ratio of 0.0042, which is coded as AY. The third material is 52CrMoV4 spring steel without pre-annealing and with a Nb ratio of 0.0376, designated as BN. Lastly, the fourth material is 52CrMoV4 spring steel, which was pre-annealed and has a Nb ratio of 0.0376, coded as BY. The flat material is designated as FM, and the final product is the leaf spring coded as LS.

**Figure 3:**
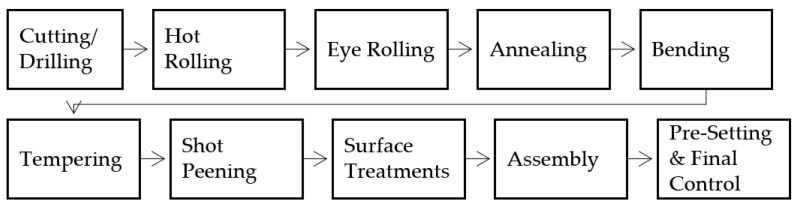
The flow chart of the production process of 52CrMoV4 spring steel.

The flow chart of the production process of the current study is shown in Figure 3. In the production of parabolic springs, after the drilling process, hot rolling was carried out to provide the raw material with parabolic thickness. The wall thickness of the steel was thinned towards the ends and brought into the desired precise tolerance sections.

The samples with/without pre-annealing were heated to 950 °C and held at this temperature for 5 min, and then, four-pass rolling was carried out by a high-stiffness, two-roller, hot rolling mill. During the hot rolling process, different pressure values were created in all regions to obtain parabolic thickness, and an approximately 55% decrease was achieved in the region with the lowest thickness for the leaf spring. It is generally advised to hot roll 52CrMoV4 steel between 850 °C and 1050 °C. Upon analysis of the investigation results, it was found that hot rolling was typically carried out at temperatures close to 900 °C [15,16]. Andrade et al. investigated 0.035 wt.% Nb heat treatment and found that Nb (C) was detected at 900 °C. In the eye rolling process, the heated intermediate product was curled from its ends and the required part for the assembly was created [11].

**Figure 4:**
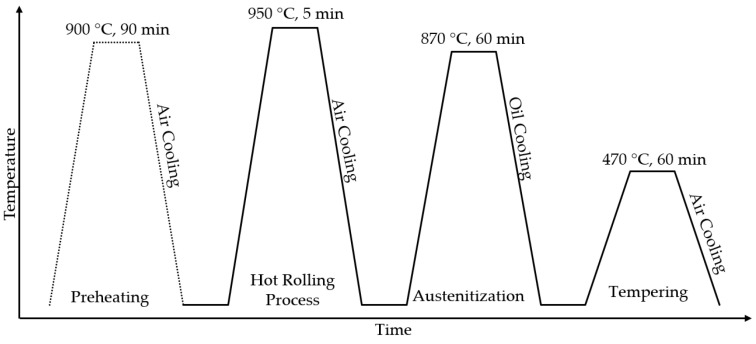
The heat treatment of the investigated materials.

The annealing process was applied to the leaf springs by heating them homogeneously within the austenite phase temperature, for example, 870 °C, and the steel samples were held at this temperature for 60 min for 52CrMoV4 steel austenization. The suggested austenite temperature for 52CrMoV4 steel is between 850 °C and 900 °C. Upon analysis of the results of other studies, it was found that temperatures between 850 °C and 870 °C are typically used for austenitization [15,17,18]. The purpose of austenitizing is to make the material suitable for both the hot forming and hardening of its internal structure. The material was bent and curved according to the desired curve and form parameters, using the hot plastic forming method. After austenization, the samples were quenched in oil at room temperature. Tempering after quenching was carried out for 60 min at 470 °C. The samples were then cooled in the air. The heat treatment process used for the materials is shown in Figure 4. After the heat treatment process, the tempering process was carried out. Next, the shot-peening process began. After sandblasting, surface treatments and assembly operations were carried out. After the final inspection, parabolic leaf spring production was complete. All tests mentioned below were performed on and compared for both flat materials and the final products.

The microstructural investigations of the samples were carried out using a scanning electron microscope (SEM) (Zeiss Microscopy, Jena, Germany) and a stereomicroscope (Leica Microsystems, Heerbrugg, Switzerland). For the microstructure investigation, the samples were prepared following conventional metallographic procedures of grinding (SiC polishing paper: 120, 220, 320, 500, and 1200 grit size) and polishing (with 0.05 μm alumina emulsion) and etching using Nital (2 vol.% solutions of nitric acid in ethanol) with an automated polishing machine. The tensile strengths of the samples were measured in accordance with the ASTM E8/E8M standard with a 250 kN capacity Shimadzu universal testing machine (Kyoto, Japan) [19]. Of note, the tensile test to determine yield strength, tensile strength, elongation, and area reduction were tested under room temperature conditions. Tensile testing was performed using a video extensometer at a speed of 3 mm/min. Based on the image segmentation results, the planimetric method was used to calculate the grain size according to ASTM E112 with a Leica Microsystem [20]. The planimetric technique counts the real grains in a predetermined area. The ASTM grain size number is calculated using the number of grains per unit area. The method’s accuracy depends on how many grains are counted. With modest effort, an accuracy level of 60.25 grain size units can be obtained. There is no bias in the results, and they are repeatable and reproducible within fewer than 60.5-grain size units. It is necessary to mark off the grains as they are counted in order to obtain an exact count. The grain size standard deviation is calculated based on the total number of grains in the field. In general, when the count per circle application and the overall count (i.e., the number of applications) grow for a particular grain structure, the standard deviation improves [20].

## 3. Results and Discussion

**Figure 5:**
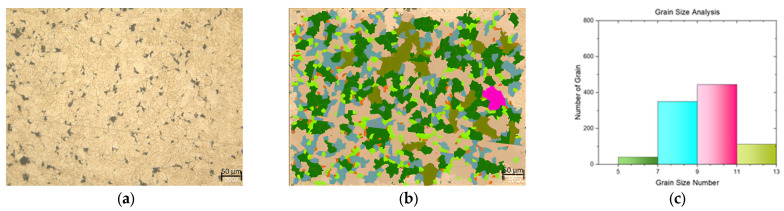
Measurement of grain size for the flat material with Nb (0.0042 wt.%) and without pre-annealing: (**a**) microstructures; (**b**) grain patterns; and (**c**) the grain size distribution according to the grain patterns.

**Figure 6:**
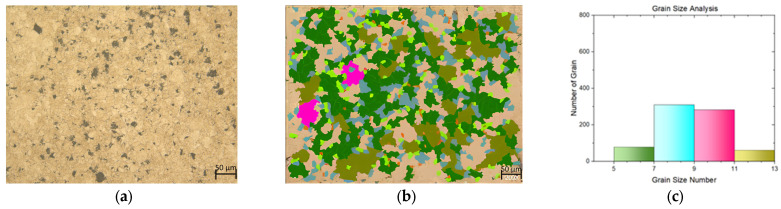
Measurement of grain size for the flat material with Nb (0.0042 wt.%) and with pre-annealing: (**a**) microstructures; (**b**) grain patterns; and (**c**) the grain size distribution according to the grain patterns.

**Figure 7:**
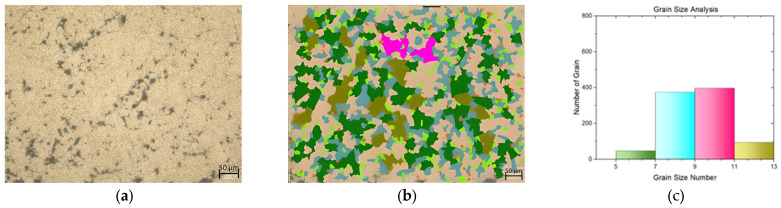
Measurement of grain size for the flat material with Nb (0.0376 wt.%) and without pre-annealing: (**a**) microstructures; (**b**) grain patterns; and (**c**) the grain size distribution according to the grain patterns.

**Figure 8:**
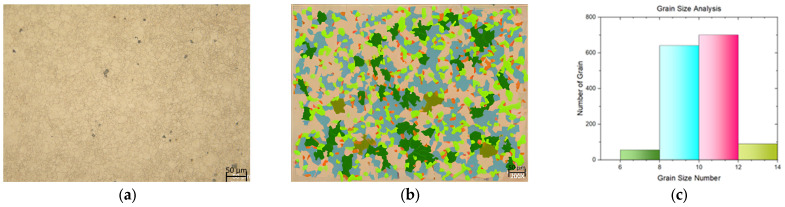
Measurement of grain size for the flat material with Nb (0.0376 wt.%) and with pre-annealing: (**a**) microstructures; (**b**) grain patterns; and (**c**) the grain size distribution according to the grain patterns.

**Figure 9:**
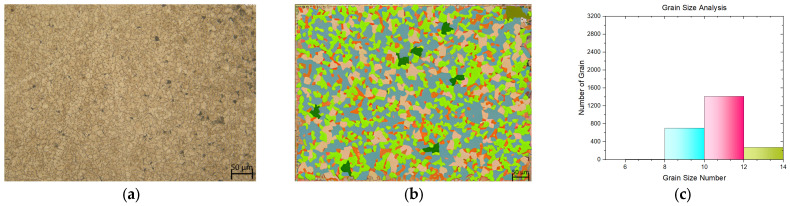
Measurement of grain size for the leaf spring with Nb (0.0042 wt.%) and without pre-annealing: (**a**) microstructures; (**b**) grain patterns; and (**c**) the grain size distribution according to the grain patterns.

**Figure 10:**
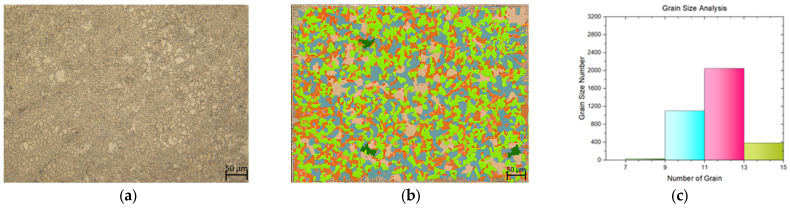
Measurement of grain size for the leaf spring with Nb (0.0042 wt.%) and with pre-annealing: (**a**) microstructures; (**b**) grain patterns; and (**c**) the grain size distribution according to the grain patterns.

**Figure 11:**
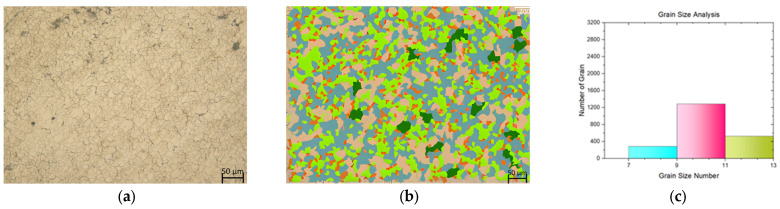
Measurement of grain size for the leaf spring with Nb (0.0376 wt.%) and without pre-annealing: (**a**) microstructures; (**b**) grain patterns; and (**c**) the grain size distribution according to the grain patterns.

**Figure 12:**
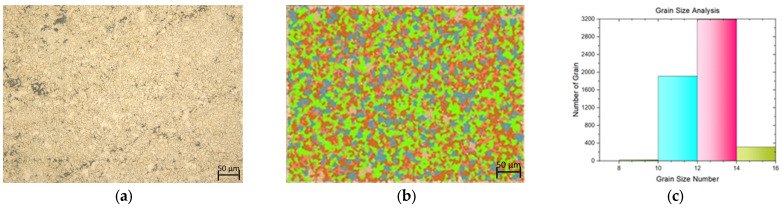
Measurement of grain size for the leaf spring with Nb (0.0376 wt.%) and with pre-annealing: (**a**) microstructures; (**b**) grain patterns; and (**c**) the grain size distribution according to the grain patterns.

**Figure 13:**
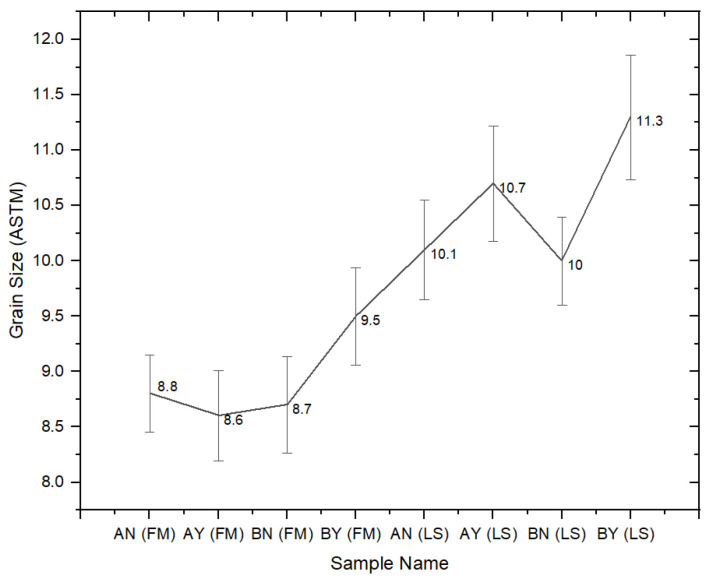
Grain size measurement with standard deviation.

**Figure 14:**
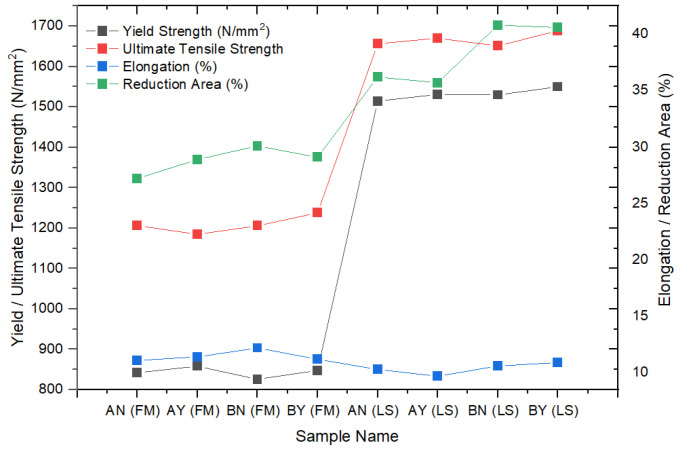
Measured mechanical properties of the investigated steels.

In this study, a tensile test was conducted on both flat materials and leaf springs produced after hot forming and heat treatment processes. While Figure 5, Figure 6, Figure 7, Figure 8, Figure 9, Figure 10, Figure 11 and Figure 12 show the image segmentation results with the microstructures, Figure 13 shows the grain size distribution and standard deviation. Figure 14 shows the tensile test results of the investigated steels.

The microstructure before heat treatment with a low Nb ratio without pre-annealing is shown in Figure 5, while the microstructure before heat treatment with a low Nb ratio pre-annealing is shown in Figure 6. The microstructure before heat treatment with a high Nb ratio without pre-annealing is shown in Figure 7, and the microstructure before heat treatment with pre-annealing and a high Nb ratio is shown in Figure 8. There appears to be an undissolved phase, as illustrated in Figure 5, Figure 6 and Figure 7. Nevertheless, the pre-annealed flat material’s examination in Figure 8 reveals that pre-annealing has the impact of reducing the number of undissolved grains at the grain boundaries. The only difference between Figure 7 and Figure 8 is the pre-annealing of the flat materials. In Figure 8, it can be clearly seen that the grain size decreases with the effect of pre-annealing. It can be seen that pre-annealing does not have any effect on the flat material with a lower Nb content (0.042 wt.%). It is understood that when the Nb ratio increases, pre-annealing is effective in reducing the grain size. When the relevant literature was examined, it was seen that the grain size decreased with the effect of Nb [5,6,7,8,9,10,11].

The microstructure following heat treatment with a low Nb ratio without pre-annealing is shown in Figure 9, and the microstructure following heat treatment with pre-annealing with a low Nb ratio is shown in Figure 10. While the effect of pre-annealing was not seen in the material with a low Nb content before heat treatment (Figure 5 and Figure 6), it was observed that the grain size of the pre-annealed material was smaller in the leaf spring material after heat treatment (Figure 9 and Figure 10). During forming, the steel may undergo phase transformations such as recrystallization, which helps in refining the grain structure [15]. The microstructure with a high Nb ratio following heat treatment without pre-annealing is shown in Figure 11, while the microstructure with a high Nb ratio following heat treatment with pre-annealing is shown in Figure 12. Likewise, it can be seen that the material with a high Nb ratio has the lowest grain size after heat treatment when pre-annealed.

The corresponding standard deviation is shown in Figure 13. According to the ASTM E112 standard, the mean linear intercept, standard deviation, 95% confidence limit, and percent relative accuracy were determined [20]. As seen in Figure 13, the standard deviation according to the ASTM grain size number was calculated as a maximum of 5%. The lowest standard deviation, at 3.45%, belonged to the flat material with a low Nb ratio without pre-annealing, while the highest standard deviation rate, at 5%, was observed in the spring material with a high Nb ratio with pre-annealing. It can be seen that the grain size of the flat material with high Nb content and pre-annealing is smaller than the other flat materials, even without the other heat treatments, due to the effect of Nb and pre-annealing, considering the standard deviations.

It is known that by using the precipitation hardening mechanism, heat treatment generally produces a microstructure with smaller grain sizes. This is the way in which the dislocation locking mechanism works in conjunction with the development of tiny phases to produce materials with improved strength characteristics [6,21]. Similar to previous research, it was discovered that the production of Nb and C particles following heat treatment results in a higher Nb ratio material with a finer grain size [5,7,8,9,10,11]. The mechanical properties of the steel are greatly affected by the size of the prior austenite grain [22].

As can be seen in Figure 13 and Figure 14, the values for tensile strength increased and the grain size decreased after the hot-forming and heat treatment process. In light of these findings, the yield strength values of the LS samples were approximately two times greater than those of the FMs. Similarly, the ultimate strength (σ_UTS_) value of the LS samples was measured at about 1650 N/mm^2^, which was higher than that of the FMs (mainly 1200 N/mm^2^). The reduction in area values, which demonstrate the level of ductility of steel materials, was measured as 36–40% in the LS samples, while the same value was measured as 28–29% in the FMs. Moreover, the approximately near elongation values were calculated as 10–11% in both groups of samples. The grain size number of the LS samples varied between 10 and 11, and the number for the other groups of samples varied between 8.5 and 9.5. It is known that when the ASTM grain size number increases, the grain size decreases, and fine-grained steels become stronger. Therefore, the ASTM grain size numbers increased from 8.6 to 9.5, while the UTS values increased from 1184 N/mm^2^ to 1237 N/mm^2^ in the FM samples. A similar increase from 10.0 to 11.3 for the LS samples resulted in an increase in the UTS from 1651 to 1688 N/mm^2^. In addition, the difference between yield and tensile strengths is great for flat materials; however, it is close in leaf spring geometry. With a low Nb content (0.0042 wt.%), the tensile properties were not affected by the pre-annealing process in both the FM samples and the LS samples. On the other hand, when the Nb alloying element content was increased to 0.0376 wt.%, there was a slight increase in the yield and tensile strengths, with them being measured at 847.7 N/mm^2^ and 1237 N/mm^2^ in the FM, respectively. A similar trend was observed in the LS samples in terms of yield and tensile strengths, with them being measured at 1550 N/mm^2^ and 1688.6 N/mm^2^, respectively. It was considered that this increment in tensile properties was not only due to the increase in Nb content but also due to the pre-annealing process. The optimum parameters to gain the highest tensile properties were seen at 0.0376 wt.% Nb content and pre-annealed conditions in the leaf spring sample. Similar results were found by Klinkenberg et al. [6]. The researchers studied the effects of adding niobium alloying elements to steels in automotive applications. The grain refining effect of niobium is mainly due to the delay or prevention of recrystallization in the final hot-forming step. The elongated grains and high dislocation density of austenite enhance ferrite nucleation. Niobium in solid solution simultaneously increases the ferrite nucleation rate and decreases the grain growth rate. This combined effect leads to a particularly fine-grained transformation structure. It is mentioned that in order to make the most of its metallurgical potential, annealing should be performed before hot working to dissolve Nb(C,N) precipitates before hot forming [6]. Klančnik et al. studied the hot deformation behavior of C-Mn steel with a wt.% 0.024 Nb microalloying element. This study showed that when comparing the initial prediction to the experimental results, a strong correlation was found in terms of Nb efficiency and how the grain size evolution affected the ductility qualities. In their investigations, they discovered that the toughness increased, and the grain size decreased with the addition of Nb [23]. Su et al. investigated the mechanical properties of carbon pearlitic steels with different rates of the Nb element. In their investigation, the inclusion of Nb increased the yield strength of the steel. The temperature of austenitization was shown to be associated with the strengthening mechanism of Nb in high-carbon pearlitic steels. Nb’s strength increased at a lower austenitization temperature (900 °C) as a result of precipitation and grain refinement strengthening [24]. The microstructure and mechanical characteristics of medium carbon bainitic steel with a weight percentage of 0.018 Nb microalloying element were examined by Zhanf et al. The greater yield strength and work hardening capacity of Nb steel may be attributed to the finer bainitic ferrite plates and the higher average carbon concentration of residual austenite [25].

**Figure 15:**
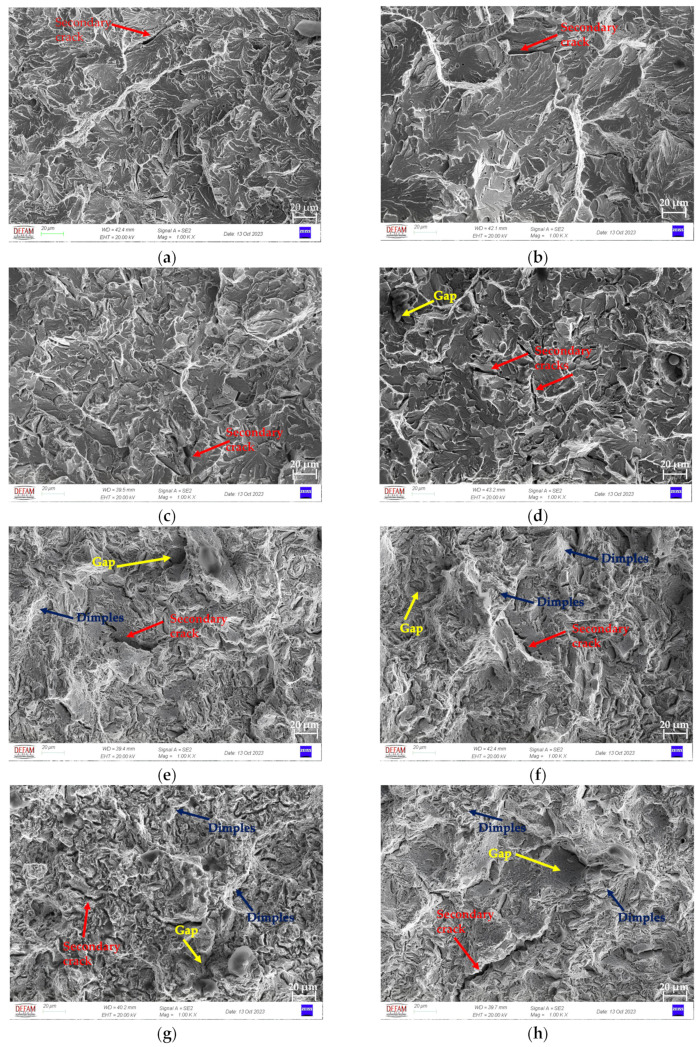
SEM images of fractured surfaces after the tensile tests: (**a**) flat material with Nb (0.0042 wt.%) and without pre-annealing; (**b**) flat material with Nb (0.0042 wt.%) and with pre-annealing; (**c**) flat material with Nb (0.0376 wt.%) and without pre-annealing; (**d**) flat material with Nb (0.0376 wt.%) and with pre-annealing; (**e**) leaf spring with Nb (0.0042 wt.%) and without pre-annealing; (**f**) leaf spring with Nb (0.0042 wt.%) and with pre-annealing; (**g**) leaf spring with Nb (0.0376 wt.%) and without pre-annealing; and (**h**) leaf spring with Nb (0.0376 wt.%) and with pre-annealing.

After the tensile tests were completed, SEM images were captured from the fractured surfaces. Figure 15 shows the SEM micrographs of the fractured surface of the tensile specimen after the tensile tests.

The images in Figure 15 show the fracture surface and the geometry of the dimples. The SEM images in Figure 15a–d display the FMs’ fracture surface and those in Figure 15e–h show the LS materials after hot forming. It can be observed that the tear valleys are deepened, and the size of the dimple increases in the leaf spring materials compared to the flat materials. The more energy consumed during fracturing, the deeper the dimple or tear valley [26,27]. For smaller grains, the movement and sliding of dislocation at the grain boundaries becomes more difficult; hence, a smaller sized pit is formed during this coordinated deformation [28]. The blue arrows shown in Figure 15e–h are given as examples of some of the dimples. It can be observed that as the number of dimples increases, a more ductile fracture structure can be seen. The smallest dimples can be observed in Figure 15h. It can be seen that there is a more ductile fracture, and when the tensile strength results are examined, it can be seen that high strength is achieved without reducing the % reduction area; that is, there is evidence of a more ductile fracture.

The LS materials showed an intergranular fracture combined with a large number of dimples along the grain faces, as shown in Figure 15e–h. Every face can be regarded as the previous austenite’s grain boundary, in line with studies in the literature [27]. It is interesting to note that a few small secondary cracks appeared, and there is a wrinkled area close to the fracture initiation point. All of these findings demonstrate that many micropore nucleation sites are activated after this heat treatment process. Figure 15 shows the gaps formed as a result of pieces breaking off from the surface in the parts indicated by the yellow arrow. LS steels have been found to undergo ductile fracture in three consecutive stages: void nucleation, void propagation, and void coalescence, which together provide a fracture surface with dimples. A ductile fracture pattern is typically produced by microvoids that are nucleated as a result of martensite necking and breaking, martensite cracking, and/or decohesion at the ferrite–martensite interface [29]. These microvoids expand within the more ductile matrix. Secondary cracks were observed in the regions indicated by the red arrow in Figure 15. The dynamic propagation of the initial crack led to high levels of stress, which in turn produced secondary cracks. These cleavage microcracks range in size from 10 to 120 μm. This implies that the propagation of a pre-existing microcrack is dependent on the applied normal tensile stress and that the cleavage fracture is propagation controlled [30].

## 4. Conclusions

The results of this investigation indicate that pre-annealing becomes necessary as the Nb content in the steel under investigation increases. The tensile properties of the material with a high Nb content and heat treatment were found to be superior to those of the other materials studied. The results also demonstrate that the addition of Nb to 52CrMoV4 steel effectively reduces the grain size. Furthermore, while the ultimate tensile strength increased, only minimal changes were observed in the cross-section value. When comparing the leaf spring materials to the flat materials, it is evident that the tear valleys are deeper and the dimple sizes are larger, indicating increased energy consumption during fracturing. A more ductile fracture structure is observed, with the smallest dimples according to the SEM images being seen in the leaf spring with a Nb ratio of 0.0376, and which are pre-annealed. Leaf spring materials exhibit intergranular fracture, with numerous dimples along grain faces, and the appearance of secondary cracks and a wrinkled area near the fracture initiation point, which indicates micropore activation after heat treatment. Overall, this study provides valuable insights into the effects of Nb content and pre-annealing on the tensile properties of parabolic leaf spring steel, which can inform future research and development efforts aimed at improving suspension system components in the automotive industry.

## Figures and Tables

**Table 1:** Chemical analysis of the samples (wt.%).

52CrMoV4		%C	%Si	%Mn	%Cr	%Mo	%V	%Nb
Standard	Min	0.48	-	0.70	0.90	0.15	0.10	-
	Max	0.56	0.40	1.10	1.20	0.30	0.20	-
52CrMoV4-A		0,53	0.28	0.98	1.12	0.18	0.13	0.0042
52CrMoV4-B		0,53	0.27	0.98	1.10	0.18	0.13	0.0376

**Table 2:** Abbreviations of the samples.

	Nb (wt.%)	Pre-Annealed
AN	0.0042	No
AY	0.0042	Yes
BN	0.0376	No
BY	0.0376	Yes

## Data Availability

Data are contained within the article.
